# Supplementary material for: Enhancing fibre-optic distributed acoustic sensing capabilities with blind near-field array signal processing
Source: Nat Commun. 2022 Jul 11;13:4019. doi: 10.1038/s41467-022-31681-x (PMC9276755; doi:10.1038/s41467-022-31681-x)
Supplement: Supplementary file 1 — Supplementary Information [file 41467_2022_31681_MOESM1_ESM.pdf]

Supplementary Information

Enhancing fibre-optic distributed acoustic sensing capabilities with blind near-field array signal processing

Felipe Muñoz and Marcelo A. Soto*

Department of Electronics Engineering, Universidad Técnica Federico Santa María, 2390123 Valparaíso, Chile

Corresponding author: marcelo.sotoh@usm.cl

Supplementary Note 1: Different DAS acoustic channel responses and measurements

The distributed feature of DAS measurements and different optical fibre orientations lead to acoustic channels with different acoustic responses and potentially measuring acoustic waves originated by distinct kinds of sources and locations[,1-3]. Supplementary Fig. 1 shows the acoustic waveforms used in this study and measured by different DAS channels along the sensing fibre illustrated in Fig. 1a, when the acoustic source is placed at the T180 position. As can be observed, all channels show very different amplitude and noise levels. This can be caused by some differences in the coupling of the optical fibre to the ground, or due to the different longitudinal strains induced by the distinct optical fibre orientations. Note that when comparing, for example, channels 190 and 200 (see Supplementary Fig. 1b), it can be seen that despite the differences in amplitude, the presence of the signal can be clearly distinguished in both channels, which means that both DAS measurement are useful for signal enhancement. However, if the amplitude difference is not corrected before combining the channels by the beamformer (e.g., delay-and-sum beamforming[4,5] as used in this work), the channel 190 (i.e., the one with larger amplitude) will dominate over the channel 200 (i.e., the one with lower amplitude), resulting in an output signal being very similar to channel 190. Therefore, it becomes very important to apply an equalisation process (see Methods) before applying any beamforming processing.

On the other hand, Supplementary Fig. 2 shows the spectrogram obtained from three different DAS example channels. The first one (Supplementary Fig. 2a) corresponds to a good-quality measurement of the chirped acoustic wave of interest in this study. The second one (Supplementary Fig. 2b) represents a measurement of the same chirped acoustic wave but contaminated by a low-frequency acoustic signal originated at a location nearby the specific DAS channel. The third case (Supplementary Fig. 2c) represents a situation where the sensing optical fibre shows a null response to the chirped acoustic wave (either because of the optical fibre orientation or a poor coupling to the ground) but shows a clear measurement of the low-frequency signal. This low-frequency wave is here considered as interference and its origin is presumably due to the mechanical vibration originated by a neighbouring highway or by the operation of a pumping plant near the study area[6,7].

DAS channels containing this low-frequency interference exhibit a lower similarity indicator κ, and must be avoided in the beamforming-based processing for signal enhancement and source location estimation. It must be indeed noted that the beamforming-based spatial filtering becomes less efficient for low frequency signals, due to the long wavelength associated, which sets low directivity conditions for beamforming spatial filtering[8]. Therefore, if some channels with this low-frequency interference are included in the processing, these frequency components will appear in the beamforming signal being generated, as illustrated in Fig. 5c, which analyses and verifies the impact of using channels with low similarity indicators.

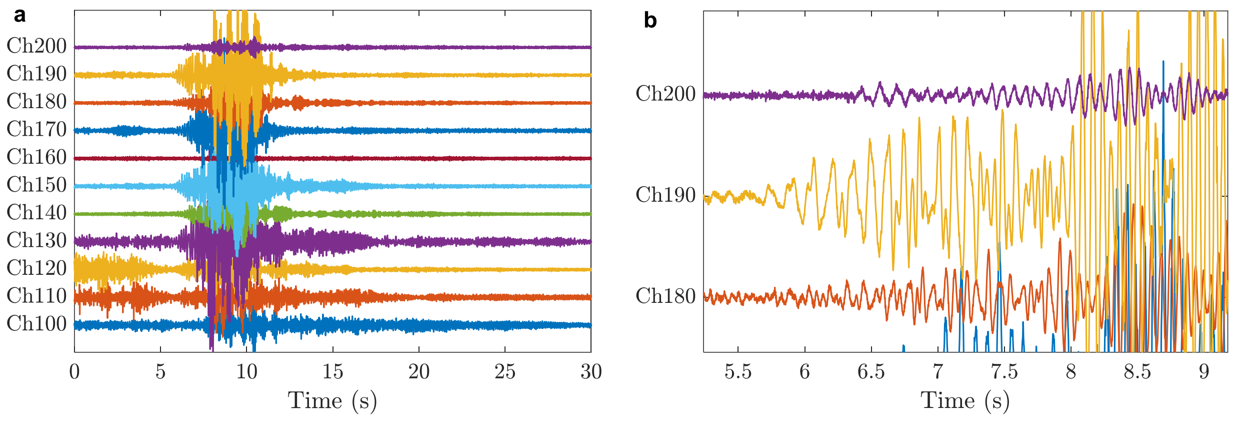

Supplementary Fig. 1. a Acoustic waveforms measured by distinct DAS acoustic channels (Ch), showing different responses to mechanical vibrations. **b** Zoom-in of three specific channels.

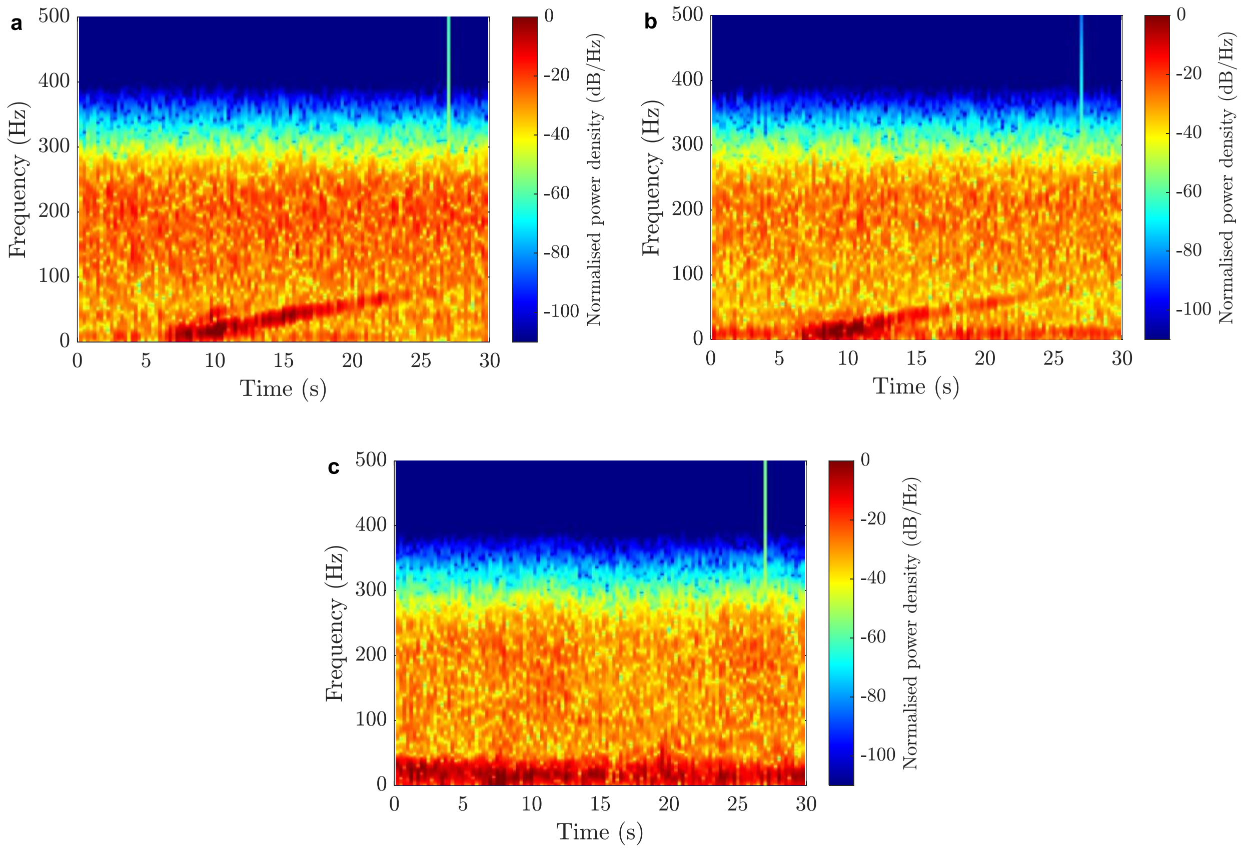

Supplementary Fig. 2. Spectrogram of acoustic wave measurements obtained by different DAS channels. Examples for: **a** a channel that clearly measures the chirped acoustic wave of interest, **b** a channel measuring the chirped acoustic wave and a low-frequency interference originated nearby, and **c** a channel with null response to the chirped acoustic wave but measuring the low-frequency interference.

Supplementary Note 2: Use of phase cross-correlation to evaluate similarity and reliability

In real-field scenarios, the acoustic waveforms acquired by a DAS system might be distorted due to ground attenuation, reflections, dispersion, and reverberations, among other possible reasons. Each channel waveform can actually be differently distorted. Distortions caused by reflections can easily generate amplitude fluctuating signals, implying that some segments of the acoustic waveform could have large amplitude, while in some moments the signal can have very low amplitude[9]. The similitude of the two channels with amplitude fluctuating waveforms can be evaluated through cross-correlation[8]. However, when using standard (amplitude) cross-correlations, large amplitude segments have greater influence on the cross-correlation amplitude, leading to high-amplitude peaks that do not necessarily represent a high similarity for that particular time delay[10,11]. A much reliable method to estimate the similarity of these kinds of waveforms is to calculate the phase cross-correlation function (PCCF) between the channels. This function is amplitude-unbiased and allows us to compare the instantaneous phases of the signals[10,11]. Therefore, the phase cross-correlation function can allow us to find the lag at which the signals have the larger number of samples with the same instantaneous phase, making it much more suitable for comparing two DAS waveforms and estimate the time difference of arrival (TDOA).

In the Methods of the paper, a procedure to estimate the similarity between channels and the reliability of TDOA estimations is presented. This procedure is based on estimating the sharpness of the PCCF using the peak-to-root mean square ratio (PRMSR)[12], which defines a similarity indicator κ. Supplementary Fig. 3 exemplifies the phase cross-correlation functions that can be obtained for a pair of DAS channels i and j under different conditions, indicating the value of the main correlation peak ρ_{ij} and similarity indicator κ_{ij}. In particular, Supplementary Fig. 3a shows a PCCF with a very sharp and clear peak, indicating good similarity between the DAS channels and also a reliable TDOA estimation. On the other hand, Supplementary Fig. 3b shows a PCCF with undistinguishable main peak, indicating the poor phase coherence and low similarity between channels. Therefore, the TDOA estimated among these two channels turns out to be highly unreliable since no clear peak is obtained, and any peak and lag obtained from the function could be wrongly estimated from noisy irrelevant correlation sidelobes. Supplementary Fig. 3c and 3d also illustrate phase cross-correlation functions with some levels of unreliability, since the first one shows to have two clear peaks, presumably due to a clear reflection affecting the measurement; while the second one shows periodic correlation sidebands caused by narrowband acoustic signals.

Note that the use of phase cross-correlation instead of amplitude cross-correlation is crucial for a reliable estimation of the level of similarity and provide reliable TDOAs. Supplementary Fig. 4 compares the impact of using a phase cross-correlation with respect to amplitude cross-correlation for TDOA estimation. Using the real source position (T180), the distance between the acoustic source and each DAS channel along the sensing fibre in Fig. 1 is calculated. Subtracting the distance between the source and the pilot trace, the differential distance to the source is plotted versus the estimated TDOA for each channel. In addition, the similarity indicator κ is calculated for each DAS channel and shown in colour scale in Supplementary Fig. 4. In Supplementary Fig. 4a, we can observe that the use of phase cross-correlation leads to estimated TDOAs that are closely placed around a straight line of slope $1/v$, with v being the propagation velocity. However, when the amplitude cross-correlation is used, Supplementary Fig. 4b shows that TDOAs are highly scattered around the straight line, indicating much lower level of reliability in the estimations. Note that in both cases, the straight line is obtained by a robust linear fit, which downplays the importance of outliers when making the fit[13]; thus, allowing for the estimation of the propagation velocity, which turns out be very similar in the two cases.

The root-mean-square error (RMSE) of the estimated TDOAs with respect to the ideal straight line is calculated as 0.029 s and 0.394 s for the phase and amplitude cross-correlations, respectively. This indicates that the use of the PCCF leads to errors in the TDOA estimations being one order of magnitude lower compared to the use of standard amplitude correlation.

Supplementary Fig. 4 also shows the reliability ranking obtained by sorting the κ value in each case, recalling that the 1st position in the ranking corresponds to the most reliable DAS channel, i.e., with the highest κ indicator, and the position 863 represents the channel with the lowest κ value. Supplementary Fig. 4a clearly shows that the blind ranking based on the PCCF helps us to discriminate the reliable estimates quite well, since the most reliable channels (blue points in the figure) are closer to the straight line.

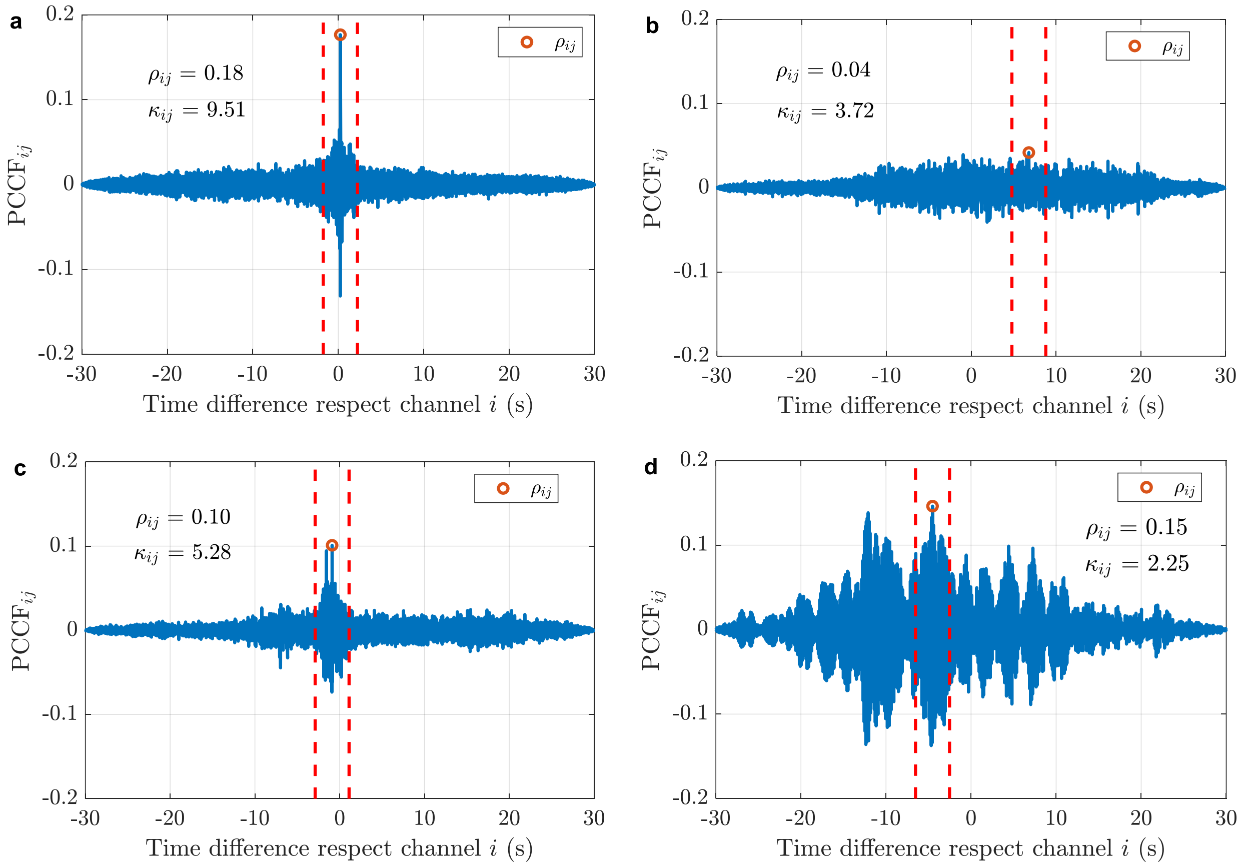

Supplementary Fig. 3. Examples of different phase cross-correlation functions (PCCF). **a** PCCF of two broadband signals, classified as similar good quality channels, with a single sharp peak. **b** PCCF of two very different waveforms, with poor phase coherence. **c** PCFF of two broadband similar signals but being affected a strong reflection, leading to two sharp correlation peaks. **d** PCFF of two narrowband signals, showing high amplitude correlation sidebands.

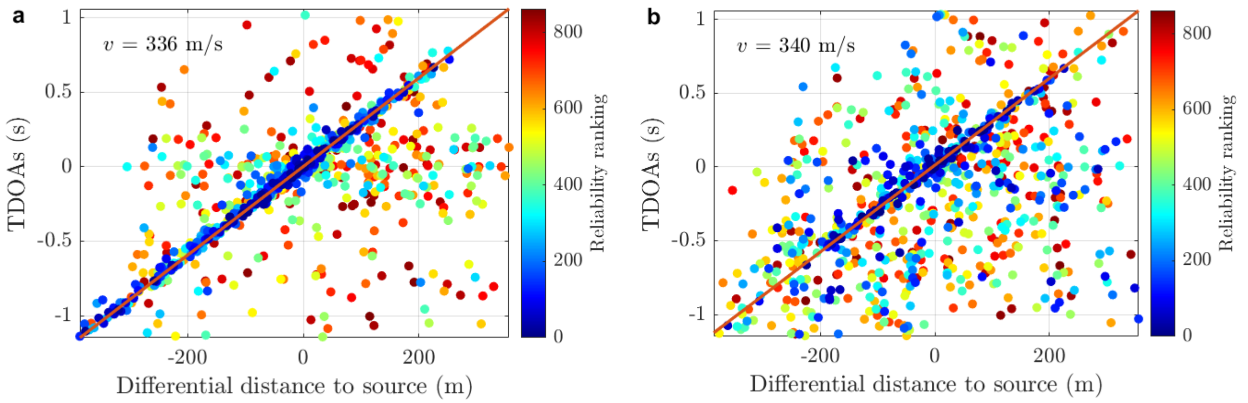

Supplementary Fig. 4. Comparison of the time difference of arrivals (TDOAs) estimated using **a** phase cross-correlation and **b** amplitude cross-correlation. The colour scale shows the ranking of DAS channels based on the blind reliability indicator κ, being 1 the most reliable estimation and 863 the less reliable one. Straight lines are obtained by a robust fitting procedure that minimises the importance of outliers. The slope allows for the estimation of the propagation velocity (v).

Supplementary Note 3: Complementary results of beamforming-based processing

Delay-and-sum spatial filtering for acoustic signal enhancement

The impact of delay-and-sum beamforming on the output signal is evaluated by calculating the normalised similarity indicator (see Methods) of this signal with respect to the reference acoustic waveform. Supplementary Fig. 5 shows the similarity indicator when DAS channels are averaged after applying trace alignment based on the estimated TDOA (i.e., when applying delay-and-sum beamforming) and when using a simple trace averaging with no temporal alignment. Results clearly point out that the use of beamforming can increase the similarity of the obtained waveform with respect to the reference, when compared to the pilot trace (first point in the figure). However, the use of simple trace averaging significantly reduces the similarity of the obtained trace with respect to the reference, even when using only a few DAS channels (note the large reduction of similarity already in the second point of the figure, representing the use of the 21st most reliable DAS channels). It can be seen that the similarity decreases rapidly when using no-aligned trace averaging, decaying practically to zero when using a large number of channels. This result highlights the importance of the time synchronisation procedure based on reliable TDOA estimations used by delay-and-sum beamforming.

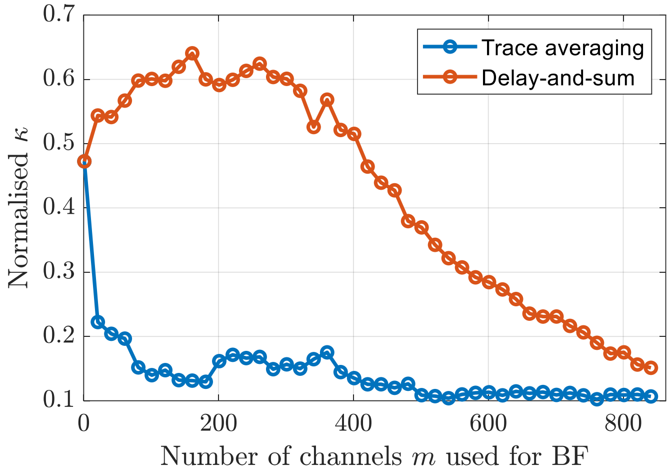

Supplementary Fig. 5. Non-blind evaluation of the similarity of the beamforming-enhanced DAS acoustic signal. Comparison of the normalised similarity indicator κ when using delay-and-sum beamforming (red curve) and simple trace averaging with no trace alignment (blue curve).

Evaluating errors in the source position estimation

Supplementary Fig. 6 shows all estimated source locations $\mathbf{X} = [\mathbf{x}_5, \mathbf{x}_h, \ldots, \mathbf{x}_{860}]$ calculated for all values of h channels used. A close-up of the area near the actual (reference) source position is shown in Supplementary Fig. 6b. Using these estimated source positions, the estimation errors in Fig. 8a are calculated. Note that from all the estimation errors depicted in Fig. 8a (ranging from 3 m up to 40 m), Supplementary Fig. 6a point out that even the larger absolute errors correspond to small relative errors compared to the distance between the acoustic source and sensing optical fibre.

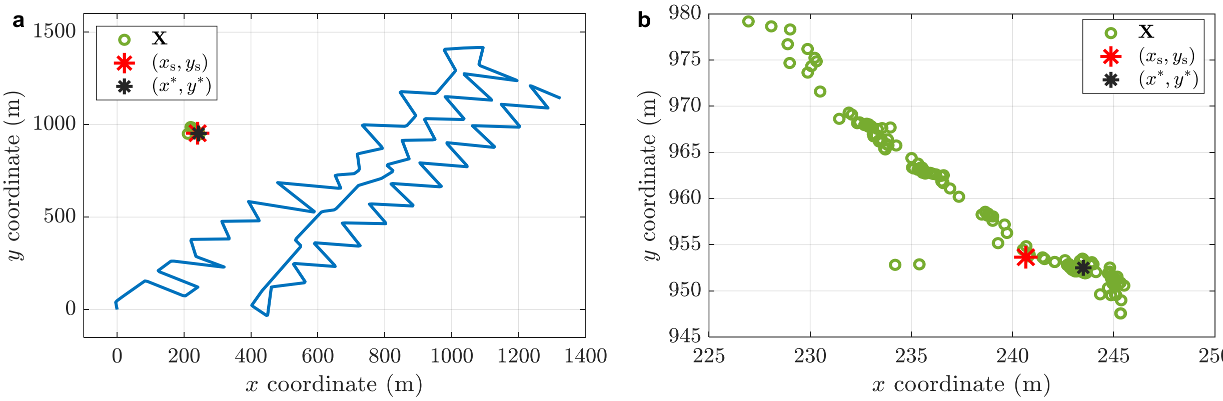

Supplementary Fig. 6. Estimated source positions using different h numbers of DAS channels. **a** Comparison of the spatial coordinates with respect to the sensing optical fibre position. **b** Zoom-in of all estimated positions, including the actual source position (red star marker), the spatial coordinates estimated by the proposed method with different values of h and histograms.

Comparing times of arrival for each DAS channel

Supplementary Fig. 7 shows the times of arrival obtained using the estimated velocity and the distance from the source to each DAS channel, where the distances are obtained using: *i)* the actual source position (x_s, y_s) and *ii)* the estimated position (x^*, y^*). Both curves are very similar to each other, indicating that by obtaining an estimation of the source position, the initially estimated TDOAs shown in Fig. 3, can be improved through an iterative process as described in the discussion of the manuscript.

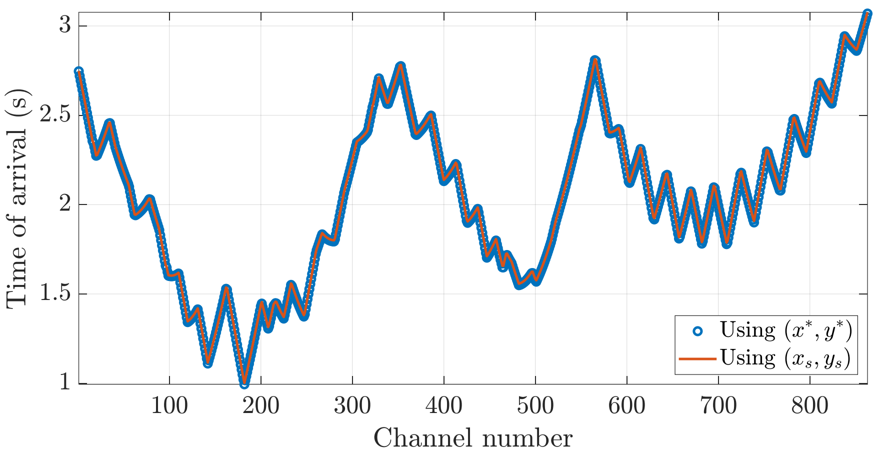

Supplementary Fig. 7. Comparison of times of arrival obtained using the estimated velocity and the distance from the acoustic source to each DAS channel, where the distances are obtained using the actual source position (x_s, y_s) and the estimated position (x^*, y^*).

Supplementary Note 4: Iterative beamforming

Supplementary Fig. 8a shows the results obtained for an example beamforming signal obtained through different iterations of the algorithm for a fixed number of channels m. Two situations are shown: *i)* using the blindly found optimal number of channels $m = m^*$, and *ii)* using all DAS channels $m = 863$. Recalling that the channels are ordered according to their reliability, the first case is an example of DAS channels with good quality signals, while the second case is an example of good quality channels mixed with bad quality ones. For each case, the normalised κ_{rl} indicator is obtained to evaluate the similarity of the resulting signal in each iteration l with respect to the reference signal r. The first point in the figure, corresponding to iteration 0, shows the value corresponding to the pilot trace, used as a reference to evaluate the improvement of similarity, which is also depicted as a red dashed line in the figure.

Results points out that in the case of using the optimal number of channels $m = m^*$, there is an improvement in the similarity until the 3rd iteration and then the beamforming signal exhibit no changes. However, when all DAS channels are used, the similarity reduces at each iteration, even below the similarity of the pilot trace, which means that the use of unreliable channels impairs the beamforming results.

To measure the changes of the estimated TDOAs between two consecutive iterations l, the indicator $\xi(l)$ is obtained from the RMSE evaluation according to:

$$\xi(l) = \sqrt{\frac{1}{m}\sum(\ \boldsymbol{\tau}^{l} - \boldsymbol{\tau}^{l-1}\ )^2} \qquad (S1)$$

where the sum is calculated for each element of the vector of TDOAs $\boldsymbol{\tau}$.

Supplementary Fig. 8b shows $\xi(l)$ for the two cases analysed. Results verify that when using m^* channels (blue curve in the figure), the TDOA estimations converge quite fast, after 3 iterations, which implies that the beamforming signal remain unchanged after 3 iterations, since the same m^* channels are aligned using the same TDOAs. On the other hand, when all DAS channels are used (orange curve), the value of $\xi(l)$ is comparatively very large (note the differences in the order of magnitude between the left-hand and right-hand vertical axes), indicating also a large variability in each iteration. These results indicate that depending on the quality and the number of channels utilised, the iterative beamforming method can either increase the similarity of the output signal or reduce it. If all the signals to be aligned are classified as good quality, the resulting signal will improve until the estimated TDOAs stop changing between successive iterations. On the other hand, if good quality signals are mixed with bad quality ones, the resulting signal will be worse, and then, the TDOAs estimated in the next iteration will also be impaired, implying that the signal and TDOAs will worsen with each iteration. Note also that the convergence obtained when using m^* channels indicates that a stopping criterion can be utilised to find the iteration at which there are no more changes in the TDOAs, and this way reduce the computational time.

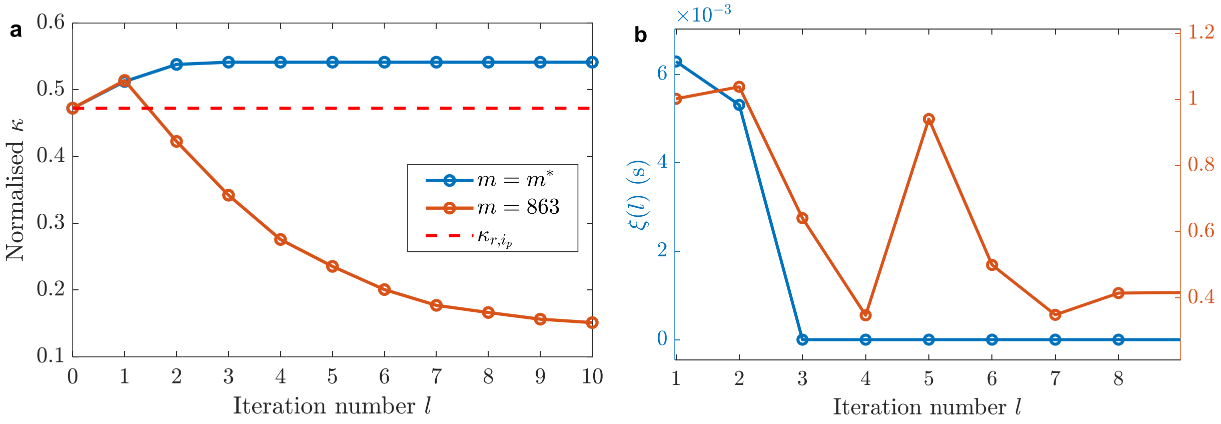

Supplementary Fig. 8. Evaluation of the iterative beamforming process, comparing the use of the optimal number of channels m^* (blue curves) and all DAS channels (orange curves). **a** Non-blind evaluation of the similarity indicator for the beamforming signal resulting at different iterations. **b** RMSE between successive estimated TDOAs at each iteration (note the different scales for both vertical scales).

Supplementary Note 5: Selecting the initial source position for searching algorithm

To evaluate the impact of the initial position used during the source location estimation[14], the searching algorithm is tested on 32 initial points surrounding the DAS channel with the largest RMS value, as shown in Supplementary Fig. 9a. These starting points belong to concentric circles with radii of 100, 200, 300 and 400 m, with 8 points per circle. The absolute estimation error $e_{h,s}$ (same as in Fig. 8a) is obtained for each starting point. The mean and standard deviation of the obtained errors are shown using error bars in Supplementary Fig. 9b. Note that the standard deviation is practically unnoticeable, except for values of h below 30, which are shown in more detail in

Supplementary Fig. 9c. Results indicate that the use of less than 30 channels makes the estimated source position to depend on the starting point, while the use of more channels makes the estimation practically unaffected by the starting location. Supplementary Fig. 9d shows the values of the standard deviation of $e_{h,s}$, which remains below 1.5 m in all cases, except for $h < 30$. This level of error can be considered negligible in this context given the large spatial dimensions involved. Therefore, as a sake of simplicity, the starting location for the minimisation algorithm described in the Methods is here selected as the DAS channel having the highest RMS value, since in a completely blind situation this channel could presumably have direct line-of-sight or eventually be closer to the acoustic source.

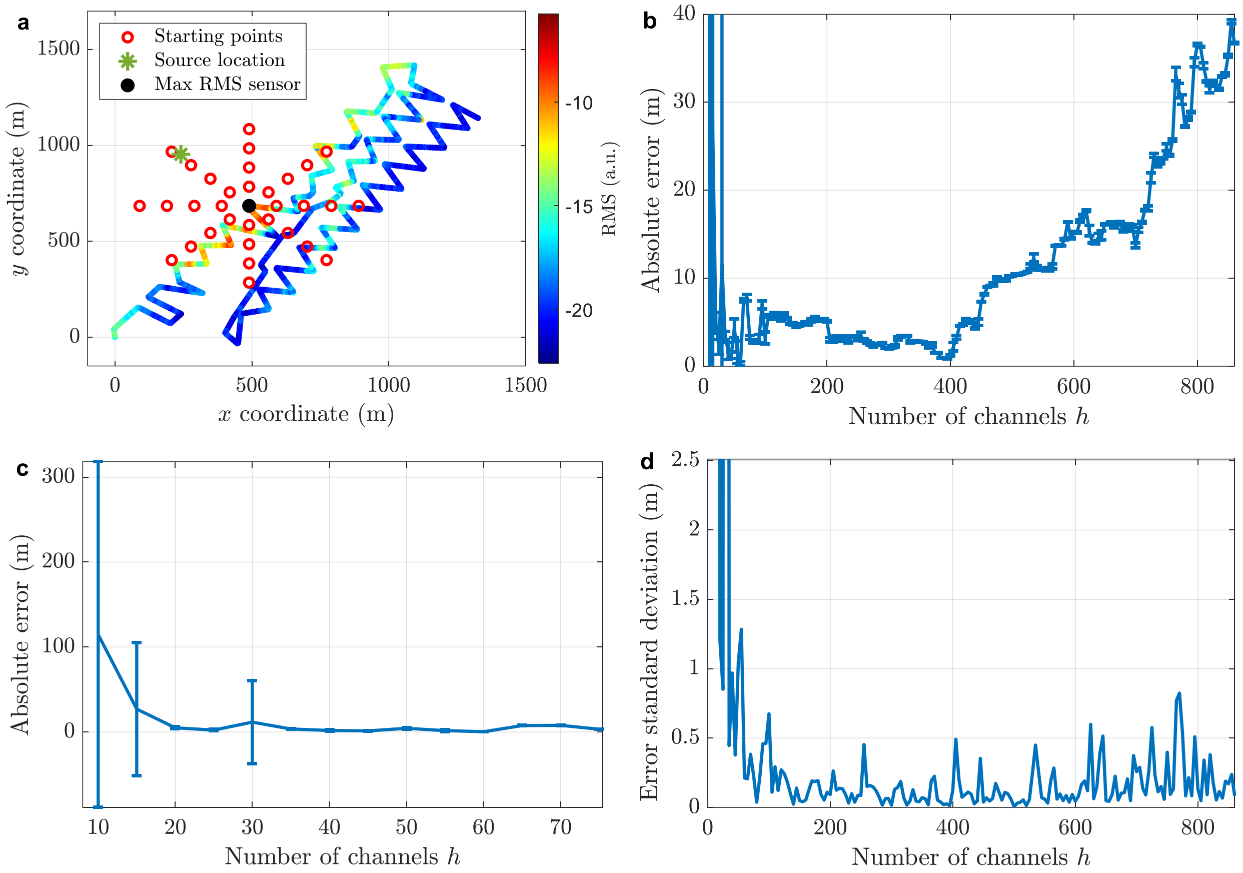

Supplementary Fig. 9. Impact of the starting point in the searching algorithm for source location estimation. **a** Positions of different initial points used for the searching algorithm, including a colour scale depicting the measurement root-mean-square (RMS) value of each DAS channel. **b** Absolute estimation error $e_{h,s}$ for all analysed starting points. The error bars represent the standard deviation of $e_{h,s}$ over the starting points. **c** Zoom-in of the absolute estimation error $e_{h,s}$ obtained for a low number of channels. **d** Standard deviation of the error $e_{h,s}$, considering all starting points, as a function of the number of channels.

´Supplementary Note 6: Statistical analysis of reliability improvement

Supplementary Fig. 10a shows the resulting similarity indicator κ after signal enhancement processing as a function of the initial indicator κ (i.e., the pilot trace similarity indicator), applied to the 50 analysed datasets. A red

dashed line of unitary slope is shown to better visualise the improvement in each case. Results points out that an improvement in the similarity of the output signal with respect to the reference acoustic waveform is achieved in almost all datasets. Supplementary Fig. 10b shows the differential improvement $\Delta\kappa$ in the similarity for each dataset as a function of the initial κ. It can be seen that the improvement exhibits high variability due to the different propagation paths existing in each dataset, highlighting the complexity of the data and propagation conditions. This means that it is not enough to have a pilot trace with good similarity, but there must also be a certain number of waveforms with high coherence in the dataset to obtain a real improvement in κ. Based on these results, the relative improvement (in percentage) can be calculated as $100\Delta\kappa/\kappa$, which is used in the horizontal axis of Fig. 9b

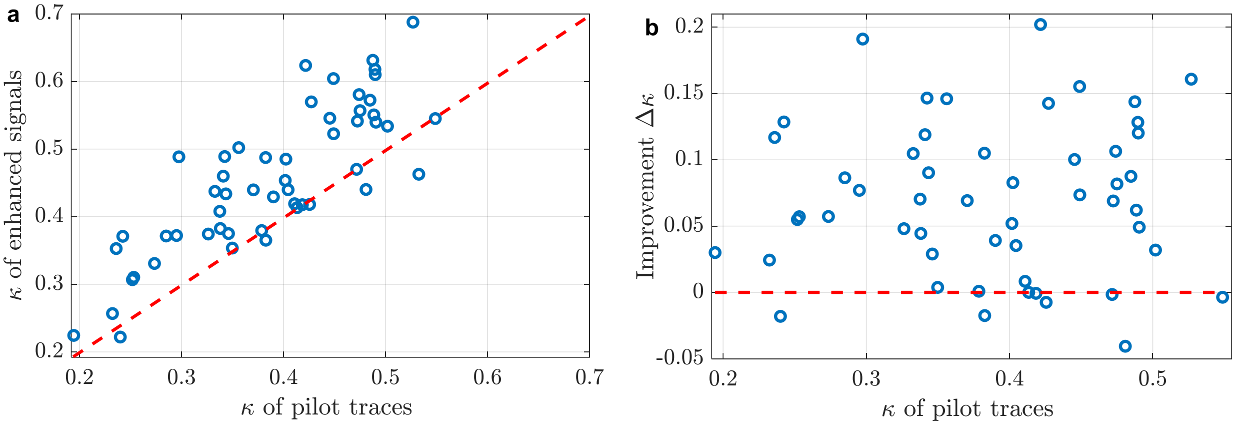

Supplementary Fig. 10. Evaluation of the similarity improvement resulting from the applied beamforming spatial filtering for the 50 analysed source location positions. **a** Similarity indicator κ of the beamforming enhanced signal as a function of the κ of the respective pilot traces. The dashed red line has unitary slope, allowing us to visualise the improvement brought by the spatial filtering. **b** Evaluation of the similarity improvement $\Delta\kappa$ versus the indicator κ of the respective pilot traces.

Supplementary Note 7: Spatial distribution of the most reliable DAS channels

Due to the DAS directivity, poor strain response of some DAS channels and the presence of multiple acoustic reflections affecting some fibre positions, only the measurements from some fibre positions are useful for array signal processing. In particular, Supplementary Fig. 11a highlights (red dots) the position of the optimal 41 channels used during the implemented beamforming-based spatial filter used for enhancing the quality of the measurements. Note that these best-quality DAS channels are randomly distributed along the sensing fibre, representing a sparse array used by the processing. In addition, Supplementary Fig. 11b and 11c show the best 100 and 200 DAS channels used during the recursive processing for source localisation based on a modified hyperbolic triangulation. The sparse (non-uniformly distributed) array is clearly composed by channels with the most reliable TDOA estimations, in agreement with the results shown in Fig. 3.

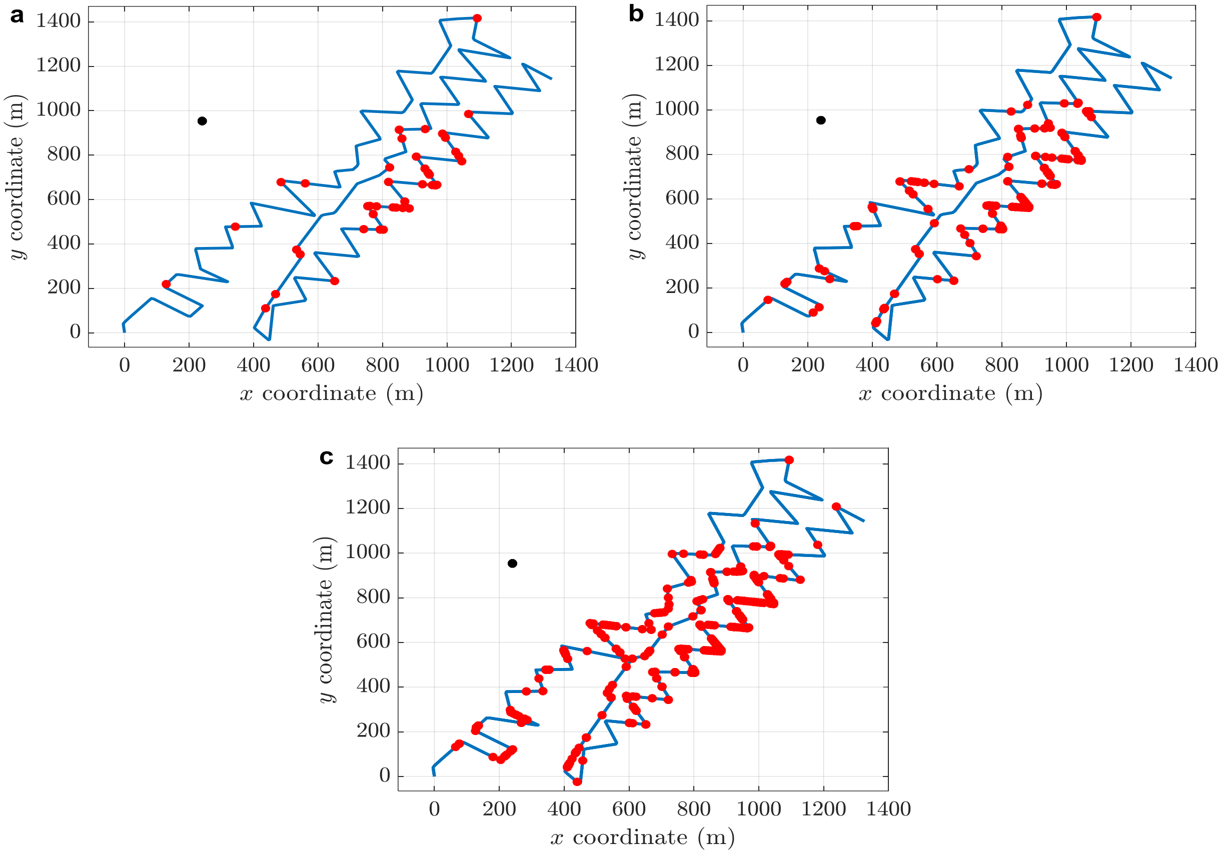

Supplementary Fig. 11. Spatial distribution of **a** the optimal 41 channels (red dots) used for the delay-and-sum beamforming for signal enhancement, **b** and **c** the 100 and 200 most reliable channels used for source localisation (note that the total number of channels in source localisation is much larger, and the plot here is limited to 200 channel only to provide visual clarity). The black dots represent the actual source location.

Supplementary Note 8: Source location error map

Supplementary Fig. 12 shows the positions of the actual source (orange circles) used in the statistical analysis, along with the position (filled coloured circle) estimated by the proposed method for each analysed dataset. The colour scale in the estimations represents the absolute error in each case. Note that, a black line connects the actual and the estimated positions, helping the visualisation of the errors in each case. The error map points out that for most of the cases, the estimated position is very close to the reference (actual) position of the source. Interestingly, small errors are obtained even when the acoustic source is located at more than 200 m away from the sensing optical fibre. The figure shows the presence of only 3 outliers, with errors over 200 m, whose estimated locations are depicted as dark red colours and linked to the actual source position through dotted lines. A further investigation is required to better understand the origin of these cases with large errors, which is presumably attributed to the local characteristics of the propagation medium.

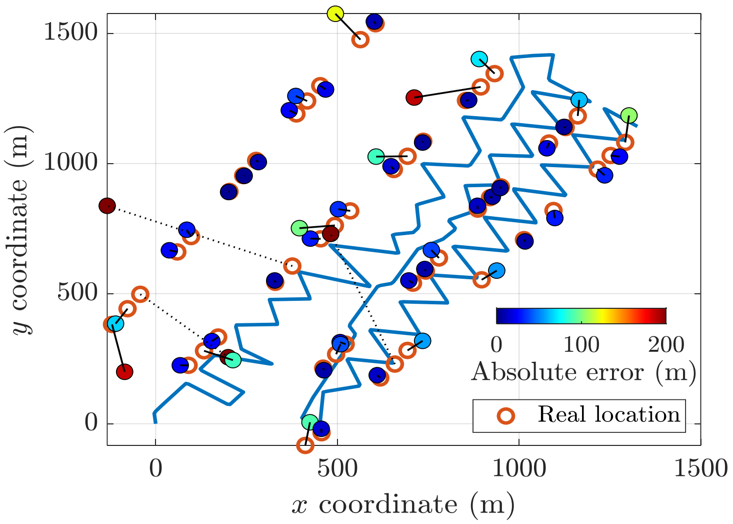

Supplementary Fig. 12. Source location error map. Real locations of the acoustic source (orange circles) are shown along with the estimated locations (coloured circles). The colour scale represents the absolute error in metres.

Supplementary References

1. Papp, B., Donno, D., Martin, J. E. & Hartog, A. H. A study of the geophysical response of distributed fibre optic acoustic sensors through laboratory-scale experiments. *Geophys. Prospect.* **65**, 1186–1204 (2017).
2. Lim Chen Ning, I. & Sava, P. High-resolution multi-component distributed acoustic sensing. *Geophys. Prospect.* **66**, 1111–1122 (2018).
3. Mousa, W. *Advanced Digital Signal Processing of Seismic Data*. Cambridge: Cambridge University Press, 2020.
4. Johnson, D. H. & Dudgeon, D. E. *Array Signal Processing: Concepts and Techniques*. Pearson, 1993.
5. Cigada, A., Ripamonti, F., Vanali, M. The delay & sum algorithm applied to microphone array measurements: Numerical analysis and experimental validation. *Mechanical Systems and Signal Processing* **21**, 2645-2664 (2007).
6. Feigl, K. L. & Parker, L.M. PoroTomo Final Technical Report: Poroelastic Tomography by Adjoint Inverse Modeling of Data from Seismology, Geodesy, and Hydrology. United States: N. p., 2019. Web. http://doi.org/10.2172/1499141.
7. Zeng, X., Thurber, C. H., Luo, Y., Matzel, E. & Porotomo Team. High-resolution shallow structure revealed with ambient noise tomography on a dense array. 42nd Workshop on Geothermal Reservoir Engineering Stanford University, Stanford, California, February 13-15, 2017, pp. SGP-TR-212.
8. Benesty, J., Chen, J. & Huang, Y. *Microphone Array Signal Processing*, Springer, 2008.
9. Mousa, W. *Advanced Digital Signal Processing of Seismic Data*. Cambridge: Cambridge University Press, 2020.
10. Schimmel, M. Phase Cross-Correlations: Design, Comparisons, and Applications. *Bull. Seismol. Soc. Am.* **89**, 1366–1378 (1999).
11. Schimmel, M., Stutzmann, E. & Gallart, J. Using instantaneous phase coherence for signal extraction from ambient noise data at a local to a global scale. *Geophys. J. Int.* **184**, 494–506 (2011).
12. Vijaya Kumar, B. V. K. & Hassebrook, L. Performance measures for correlation filters. *Appl. Opt.* **29**, 2997-3006 (1990).

13. Huber, P. J. *Robust Statistics*. Hoboken, NJ: John Wiley & Sons, Inc., 1981.
14. Li, X., Deng, Z. D., Rauchenstein, L. T. & Carlson, T. J. Contributed Review: Source-localization algorithms and applications using time of arrival and time difference of arrival measurements. *Rev. Sci. Instrum.* **87**, 041502 (2016).
